# Traditional Chinese Medicine Huannao Yicong Decoction Extract Decreases Tau Hyperphosphorylation in the Brain of Alzheimer's Disease Model Rats Induced by A*β*_1–42_

**DOI:** 10.1155/2016/6840432

**Published:** 2016-11-29

**Authors:** Yu Cao, Xingxing Jia, Yun Wei, Meixia Liu, Jiangang Liu, Hao Li

**Affiliations:** Institute of Geriatrics, Xiyuan Hospital, China Academy of Chinese Medical Sciences, Beijing 100091, China

## Abstract

*Objective*. Huannao Yicong Decoction (HYD, *还脑益聪*方) has been shown to improve the learning and memory capabilities of Alzheimer's disease (AD) subjects. However, the underlying mechanism remains to be determined.* Methods*. Sixty Sprague-Dawley rats were divided equally and randomly into five different groups including control, positive control, and HYD granules of low dose, medium dose, and high dose by daily gavage. The sham-treated rats were also given the same volume of sterile water by gavage. Twelve SD rats were treated with the same amount of physiological saline. Twelve weeks later, learning and memory capabilities, A*β* content of the right brain and the expression of glycogen synthase kinase-3*β* (GSK-3*β*), total tau protein kinase (TTBK1), and cyclin-dependent kinase-5 (CDK-5) were tested.* Results*. Our results showed that high dose HYD treatment significantly improved the learning and memory capability of the AD rats and decreased the expression of TTBK1, GSK-3*β*, and CDK-5 in the hippocampal CA1 region.* Conclusions*. HYD treatment for 12 weeks significantly improved spatial learning and memory and effectively inhibited A*β* deposition, likely via reducing tau protein kinase expression and thus tau hyperphosphorylation and inflammatory injury. Taken together, these results suggest that HYD could be an effective treatment for AD.

## 1. Introduction

Alzheimer's disease (AD) is a neurodegenerative disease, characterized by progressive memory loss and cognitive dysfunction. Intracellular neurofibrillary tangles (NFTs), extracellular senile plaque (SP), and abnormal cholinergic transmitter metabolism in the central nervous system (CNS) are pathological features observed in AD patients upon autopsy [1]. Hyperphosphorylation of tau is believed to account for most NFTs. In the brain of AD patients, the amount of total tau protein was far greater than what is typically observed in unaffected individuals, largely driven by dramatic increases in the amount of hyperphosphorylated tau [2]. At present it is well accepted that extracellular deposition of amyloid protein (A*β*) may trigger AD [3]. As tau phosphorylation correlates well with AD severity and extent of cognition impairment, investigations into improved understanding of tau and in particular hyperphosphorylated tau have attracted great attention. Currently, there is no effective treatment for AD. Recent studies have highlighted a potential for HYD in improving the clinical outcomes of old patients with mild cognitive impairment (MCI) [4]. HYD is based on the mechanism of deficiency-congestion-phlegm-toxic in Chinese medicine, which has been shown to improve metabolism disorder of free radicals, reducing inflammatory immune response and regulating cholesterol metabolism [5]. Moreover, animal models have revealed the impact of HYD on cognition impairment by decreasing A*β*-associated proteins and inflammatory immune response and inhibiting apoptosis of hippocampal neurons to maintain normal brain function [4].

In the present study, we studied the effect of HYD on spatial learning and memory in A*β*_1–42_ induced AD rats. To enhance our understanding of the mechanisms through which HYD influences AD, we also evaluated the changes in tau protein kinase expression in the hippocampus following HYD treatment.

## 2. Methods

### 2.1. Animals

All animal work described in the present study was reviewed and approved by the Committee on Ethics of Animal Experiments of Xiyuan Hospital of China Academy of Chinese Medical Sciences (Permit Number: 2011XLA10-05). A total of 72 Sprague- Dawley (SD) rats (half male and half female) at 3 months of age, weighing 200–220 g, were obtained from Beijing Vital River Laboratory Animal Technology Co., Ltd., with certificate of conformity: SCXK (Beijing) 2006-0009. The rats were raised in specific pathogen-free environment with temperature of 22–25°C, humidity of 50–70%, and a 12 h light cycle. All rats were given seven days of adaptive feeding to acclimatize.

*Establishment of Animal Model and Drug Administration*. Sixty rats were randomly selected for A*β*_1–42_ treatment. The remaining 12 rats represented the sham group and were treated with an equivalent volume of water. Prior to experimentation, A*β*_1–42_ was diluted with sterile saline to a concentration of 1 *μ*g/*μ*L and placed at 37°C for 72 h. For the A*β*_1–42_ or sham treatment, rats were anesthetized by 4% chloral hydrate at 0.9 mL/100 g body weight and fixed on the stereotaxic instrument. The head was shaved around the fontanelle region and disinfected with 75% alcohol. A 1.5 cm longitudinal incision was made using surgical scissors. Referring to brain stereotaxic instrument map of rats by Zhuge Qichuan, the bilateral hippocampal CA1 region in the dorsal part of dentate gyrus was identified. Accurate positioning was made and marked by the stereotaxic instrument (BW-SAD902 digital explicit stereotaxic instrument, Stoelting, USA): 3 mm after anterior fontanelle, 2 mm next to the midline of the left and right brain, and 4 mm under the surface of the skull. Using a dental embossed ball drill (number 10), two holes were drilled. Five microliters of condensed A*β*_1–42_ was injected slowly into each side of the hippocampal CA1 region at a speed of 1 *μ*L/min by micro flow rate pump (TJ-2A/L0107-2A-Micro Flow Rate Syringe Pump), manufactured by Baoding Longer Precision Pump Co., Ltd. To ensure an adequate diffusion, the needle was kept in the brain for 5 min and withdrawn slowly. Yunnan Baiyao was applied to the wound to protect it from infection and bleeding, and the skin was sutured. The sham group was similarly operated on but sterile saline rather than condensed A*β*_1–42_ was injected. The rats received intramuscular injections of penicillin 40000 U/day for 3 days to prevent infection and the wound was checked regularly.

The 60 AD model rats were divided into 5 groups (control, DPG, HLG, HYD-mid, and HYD-high) based on the results of initial Morris water maze test and their body weight using stratified random method with the SPSS software. The five groups received a daily gavage for 12 weeks with distilled water (water), Donepezil hydrochloride tablets suspension (0.49 mg/kg) (Donepezil), and HYD at three doses (in g crude drug/kg): 3.78 (HYD-L), 7.56 (HYD-M), and 18.90 (HYD-H), respectively. The sham group (control) received intragastric administration with distilled water. All six groups received the same total volume. During the twelve-week period, one rat from the HYD-M group died due to an accident and thus was removed from the study.

### 2.2. Preparation of HYD Extract and Donepezil Suspension

HYD granules, composed of* Radix Polygoni Multiflori*,* Radix Ginseng*,* Rhizoma Ligusticum wallichii*,* Rhizoma Acori Graminei*, and* Rhizoma Coptis *with a weight ratio of 2.4 : 2 : 1.8 : 1.2 : 1 were extracted with ethanol and water. The extract was concentrated under reduced pressure and then mixed with volatile oil of* Acorus tatarinowii Schott Naphtha*. The thick extract, prepared by the Drug Manufacturing Facility at Beijing University of Chinese Medicine, contained crude drug at a concentration of 3.2 g/kg. The extract was diluted with distilled water at designed ratios before being given to rats by gavage. Donepezil hydrochloride tablets (Aricept, 5 mg, National Medicine Permit: H20050978, lot number: 100609A), produced by Eisai China Inc., were crushed and mixed with distilled water as a suspension.

*Other Reagents*. A*β*_1–42_ (lot number bs-0107b) was purchased from Sigma-Aldrich, USA. *β*-amyloid (*β*-AP) kit (batch number: 20111015) was obtained from Beijing Huaying Institute of Biotechnology. The antibodies for brain-derived tau protein kinase (TTBK1/BDTK, phosphorylated, batch number: 909902W), glucose synthase kinase-3*β* (GSK-3*β*, lot number 110267), and cyclin-dependent kinase 5 (CDK5, batch number: 110218) were obtained from Beijing Biosynthesis Biotechnology Co., Ltd. Polink-2 plus Polymer HRP Detection System (nonbiotinylated) (lot number: K116616D) and 3,3′-diaminobenzidine (DAB) chromogenic kit (lot number: K116610D) were purchased from Beijing Zhongshan Golden Bridge Biotechnology Co., Ltd.

### 2.3. Behavior Test

Morris water maze (DMS-2 type, produced by the Institute of Materia Medica, Chinese Academy of Medical Sciences) was used before and after drug treatment to detect changes in cognitive function [6]. The test consisted of two parts: a place navigation and spatial probe test. The place navigation was evaluated daily for 4.5 days. The water in the maze was 30 cm deep and approximately 1 cm above the platform surface, which was located in fourth quadrant, with a temperature of 25 ± 1°C. During the test, an appropriate amount of ink was mixed in the water so as to turn the color opaque black. This test was conducted in a soundproof room. The procedure for this testing involved the following: (1) choosing and marking a point of pool wall from 1/2 radians of the second quadrant, (2) placing the rats against the pool wall into the water on the marked point, and (3) recording the time required to find and climb the platform (such that all limbs were on the platform). Together this procedure was called platform-locating latency (PLL). If the rats could not find the platform in 180 s, the conductor guided the rat appropriately. For the first 4 days, only PLL was recorded. On the fifth day, both PLL and swimming distance were recorded. Moreover, on the fifth day (in the afternoon), the platform was removed to perform a spatial probe test. In this test the rats were made to face the pool wall and enter the water from a random point of the second quadrant. The camera system automatically recorded the location where the platform was located as well as the time and distance of swimming to evaluate their learning and memory ability.

### 2.4. Preparation of Brain Tissue Sample

After the final Morris water maze test, six rats selected randomly from each group were anesthetized and sacrificed by decapitation. The brain tissue was immediately placed on ice. The left side of the brain was fixed in 4% neutral paraformaldehyde. The other side of the brain was used to dissect out the hippocampus and cortex. Hippocampal tissue was weighed, and 0.3 g cortex was homogenized with 2 mL of ice cold buffer and centrifuged at 2000 rpm for 10 min. The supernatant was stored at −80°C for future use.

### 2.5. Determination of A*β* by Radioimmunoassay

Radioimmunoassay was used to determine the content of A*β* in the right brain of the rats [7]. Briefly, the *β*-AP antibody was added into the brain tissue homogenate, which was labeled with ^125^I. After 2 hours, the secondary antibody was added to isolate antigen-antibody complex. The radioactivity (B) of the samples was measured together with the standards using the automatic *γ*-counting instrument (GC-911, China Science and Technology University of Industry Company, Ltd.).

### 2.6. Immunohistochemistry

The fixed brain tissue was paraffin embedded and cut into 5 *μ*m sections. H&E and Congo red staining [8] were performed to evaluate histopathological changes in the hippocampus CA1 region of the rat brains. Five rats from each group were randomly selected for brain sections. The expression of TTBK1, GSK-3*β*, and CDK-5 in brain tissue was examined using a two-step nonbiotinylated immunohistochemistry, according to the manufacturer's specifications. Briefly, 5 *μ*m brain sections were dewaxed, incubated with 3% H_2_O_2_ deionized water for 5–10 min to block endogenous peroxidase activity, washed 3 times in phosphate buffered saline (PBS) (2 minutes each), and then incubated with the primary antibodies (TTBK1, GSK-3*β*, and CDK-5; 1 : 400 dilution) at 37°C for 1 h. The slides were washed 3 times again in PBS (2 mins/wash) and incubated with rabbit anti-goat IgG secondary antibody for 30 min at room temperature. DAB chromogenic reagent was used to develop the image and the reaction time was determined by observing the tissue under microscope. The tissue was washed with distilled water, stained with hematoxylin, ethanol-dehydrated, and mounted with neutral gum. The stained sections were imaged with DpxView Pro (DeltaPix, Denmark). Three different images of the CA1 region on each section were taken, and a total of 15 images from each group were collected. The protein expression was determined by measuring the integrated optical density (IOD) with Image-Pro Plus 6.0 software.

### 2.7. Statistics

SPSS13.0 statistical package was used for data entry and processing. The data were expressed as mean ± SD. One-way ANOVA was used for the intergroup comparisons. LSD and Dunnett's* C* test were taken for heterogeneity of variance. A statistical significance was defined as *P* < 0.05.

## 3. Results

### 3.1. Effects of HYD on Spatial Learning and Memory Ability of A*β*-Induced AD Rats

**Figure 1:**
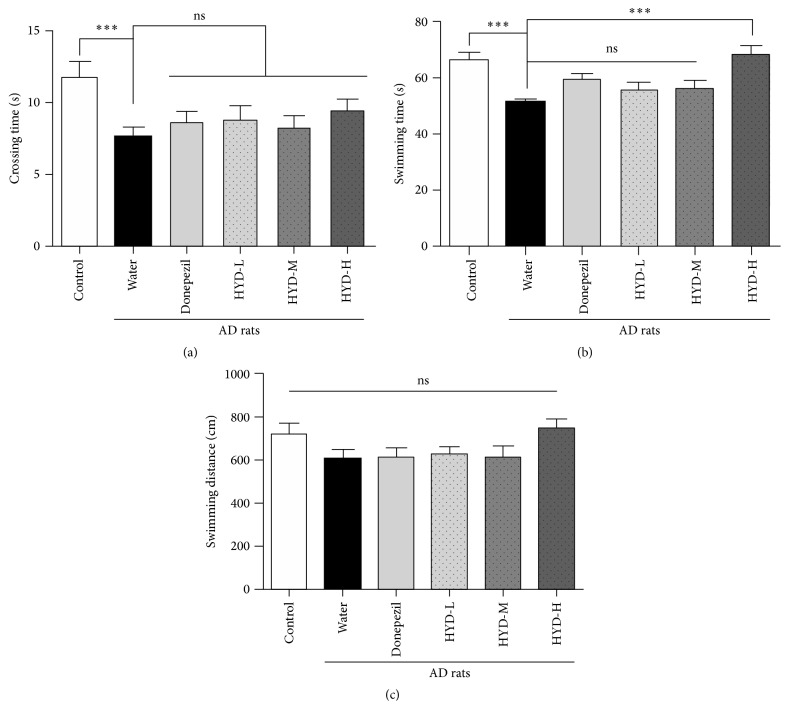
Effect of HYD treatment on learning and memory capability of AD rats. Control: sham treated; AD rats were fed with water, Donepezil, and HYD at low (HYD-L), medium (HYD-M), and high (HYD-H) dose, respectively, by daily gavage for 12 weeks.

Compared with the sham group, the AD rats had a significant decrease in the number of rats passing through the platform and the swimming time in the fourth quadrant (Figures 1(a) and 1(b)). The swimming distance of the AD rats in fourth quadrant also showed a trend to decrease although the difference was not statistically significant between the two groups (Figure 1(c)). Donepezil treatment group showed a trend of improvement (e.g., increased the number of passes and the swimming time) but such improvement was not statistically significant (Figures 1(a)–1(c)). Treatment with the low or medium dose of HYD yielded similar results as Donepezil; however, the high dose HYD treatment (18.9 g crude drug/kg) outperformed Donepezil by increasing the swimming time significantly in the fourth quadrant to that in the control rats (Figures 1(a)–1(c)). These data suggest that treatment of the AD rats with high dose HYD could improve the learning and memory abilities of these animals.

### 3.2. Effects of HYD on the Histopathology of Hippocampal CA1 Region in the AD Rats

**Figure 2:**
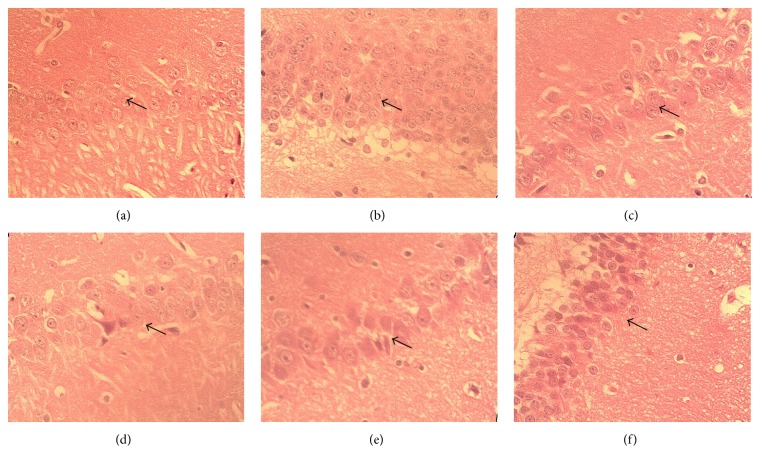
H&E staining (×600) of brain sections from the sham and AD rats. (a) Sham group; (b–f) AD rats fed with water (b), Donepezil (c), and HYD at low (d), medium (e), and high (f) dose, respectively, by daily gavage for 12 weeks.

In control rats, the cells in hippocampal CA1 region were lightly stained for intracellular structures and nucleus (Figure 2(a)). The cells were arranged in an organized fashion with clear boundaries. In contrast, the cells of the AD rats were stained much darker with expanded cytoplasm and darker pyknotic nuclei (Figure 2(b)). The cells were arranged in an irregular pattern as compared to the sham group with more randomly located cells. Following twelve weeks of treatment with HYD, the histopathology of the AD rats in hippocampal CA1 region showed various degrees of improvement, similar to the positive Donepezil treatment group (Figures 2(c)–2(f)). The area of cytoplasm and the number of darkly stained cells decreased as compared with untreated AD rats. The cells appeared to be more organized and the deep stained nucleus appeared lighter.

### 3.3. Effects of HYD on A*β* Deposition in Hippocampal CA1 Region of the AD Rats

**Figure 3:**
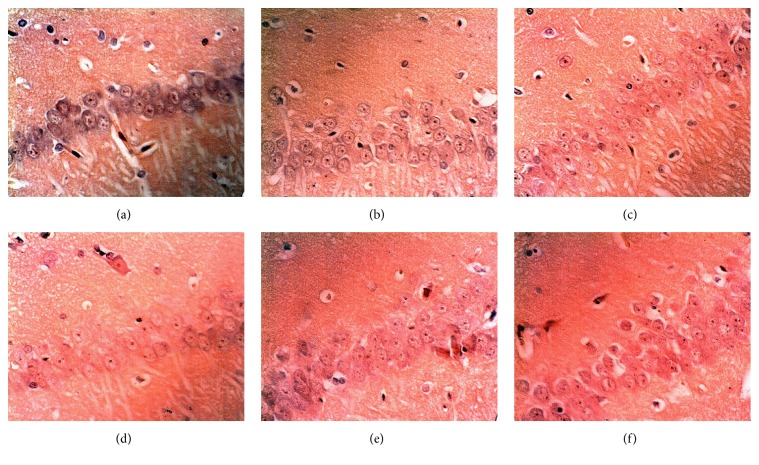
Effect of HYD on A*β* deposition in hippocampal CA1 region of the AD rats (Congo red staining, ×600). (a) Sham group; (b–f) AD rats fed with water (b), Donepezil (c), and HYD at low (d), medium (e), and high (f) dose, respectively, by daily gavage for 12 weeks.

**Figure 4:**
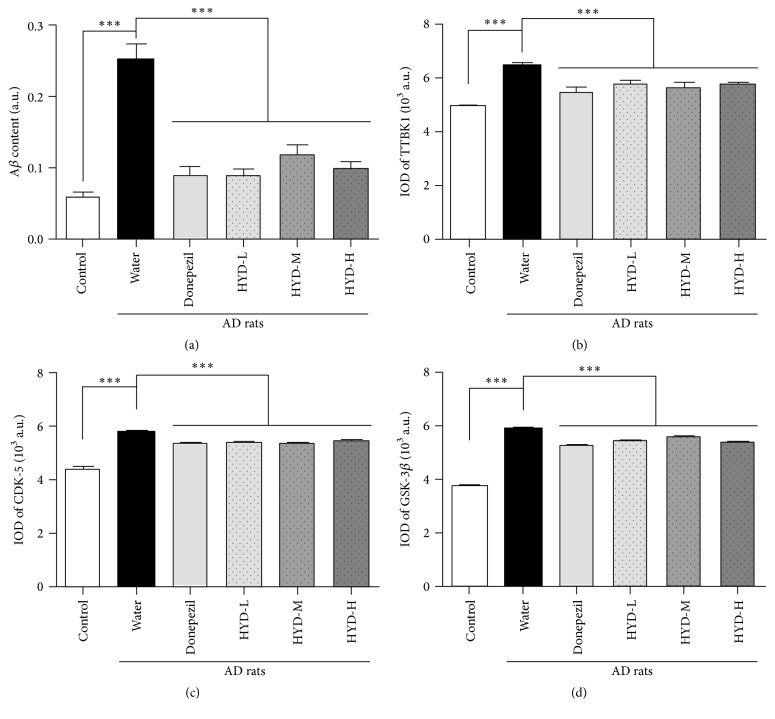
Effect of HYD on A*β* content and expression of TTBK1, CDK-5, and GSK-3*β* in hippocampal CA1 region of the AD rats.

To determine the A*β* deposition, we performed Congo red staining on the brain sections. The amount of orange amyloid deposition in the AD rats appeared to be increased as compared to sham controls and a scattered cell organization pattern was observed (Figures 3(a) and 3(b)). Following HYD treatment at three different doses or Donepezil treatment, the cells were stained lighter with clear cellular structure as compared to the untreated AD controls (Figures 3(c)–3(f)). The cytoplasm and nucleus were more clearly defined and both fewer and lighter orange amyloid deposits were observed. Consistently, the A*β* content in the hippocampus of the AD rats was significantly increased as compared with the sham group (Figure 4(a)), while all treated groups (including those treated with Donepezil and all 3 different doses of HYD) had significantly reduced A*β* content in the hippocampus (Figure 4(a)).

### 3.4. Effects of HYD on the Expression of TTBK1, CDK-5, and GSK-3*β* in the Hippocampal CA1 Region

To test whether HYD treatment has any effect on the tau phosphorylation kinases, we performed immunohistochemistry to quantify the expression of tau phosphorylation kinases. Compared with the sham group, the expressions of TTBK1, CDK-5, and GSK- 3*β* staining in the brain sections of the AD rats were all significantly increased (*P* < 0.001) (Figures 4(b)–4(d)). Importantly, HYD treatment significantly decreased the expression of these proteins, similar to that in the positive control group treated with Donepezil (Figures 4(b)–4(d)).

## 4. Discussion

The current prevailing hypothesis of AD suggests that A*β* plays a critical role in the initiation and progression of this disease. According to amyloid cascade hypothesis, A*β* acts as a trigger in both familial and sporadic forms of AD [9]. As such, it may be effective to treat or inhibit A*β* at an early stage of the pathogenesis of AD. However, a growing number of studies have shown that once AD had been triggered, tau protein was more relevant with the severity of disease [10]. These findings have led investigators to characterize the relationship between tau protein and A*β* in AD. Moreover, it was found that A*β* depositions were cleared first and subsequently reemerged prior to the tau pathology, indicating a hierarchical and direct relationship between A*β* and tau [11].

Hyperphosphorylated tau protein is a major component of NFTs. The phosphorylation level of tau protein depends on the relative activity of protein kinases and protein phosphatases. Increased A*β* could induce tau protein phosphorylation by disrupting the balance of kinases and phosphatases. The kinases involved in the phosphorylation of tau protein include GSK-3 and CDK5 [12, 13]. Interestingly, in a* Drosophila* model of AD, aberrant expression of GSK-3 and inhibition of GSK-3 both could significantly improve A*β*-induced neurotoxicity [14]. Similarly, tau defects in* Drosophila* were associated with some degree of improvement. These results indicate that GSK-3 may represent a common pathway linking A*β* and tau proteins. GSK-3 inhibition could therefore reduce the deposition of A*β* and tau protein phosphorylation [15]. Other studies have demonstrated that CDK-5 could inactivate GSK-3*β* and exacerbate the pathological process of NFT [16].

The AD rats demonstrated a decreased ability to find the platform in Morris water maze and, as compared with the sham group, brain tissue sections of the AD group showed obvious inflammatory lesions, increased cytoplasm to nucleus ratio, nucleus pyknosis, and an overall random cellular organization. Congo red staining identified an increased frequency of dark orange deposition of amyloid plaques in hippocampal CA1 region of model rats (i.e., A*β* deposition) as compared with the sham group. The A*β* content in the AD group also increased significantly (*P* < 0.01) as determined by radioimmunoassay. Following HYD treatment, the cognition expression in behavior was significantly improved and the severity of brain tissue pathology had been ameliorated. These results suggest that HYD repaired the cognitive impairment and neuronal pathological damage and may suppress A*β* formation and aggregation.

By immunohistochemistry, we evaluated the expression of TTBK1, GSK-3*β*, and CDK-5 in hippocampal CA1 regions. All three proteins were significantly increased in the AD rats as compared to the sham group (*P* < 0.01). These data suggest that A*β* affects tau protein phosphorylation, likely via inducing tau protein kinase expression. This is consistent with previous work by Tokutake et al. showing that A*β* could induce tau phosphorylation at the cellular level, and GSK-3-mediated pathway may be a gatekeeper linking these two proteins [17]. Similarly, Chabrier et al. have shown that, by injecting APP transgenic mice with low dose of A*β*, total tau protein and tau protein phosphorylation levels increased [18]. In the present study, we demonstrate that intragastric HYD treatment significantly decreased the total amount of A*β* as well as the tau protein kinase GSK-3 and CDK-5 expression, indicating that the traditional Chinese medicine prescription HYD could reduce not only the amount of A*β* but also the expression of tau protein kinases, thereby inhibiting the phosphorylation of tau proteins. As such, the traditional Chinese medicine HYD holds a great potential for the treatment of AD.

In traditional Chinese medicine, treating dementia was based on an understanding of the mechanism of deficiency-congestion-phlegm-toxic. HYD is in line with this understanding of the pathogenesis of dementia. High performance liquid chromatography was used to determine the ingredients of the HYD extract (see Figure S1 in Supplementary Material available online at http://dx.doi.org/10.1155/2016/6840432), which was found to contain seven major active ingredients: emodin, stilbene glycoside, ginsenoside Re, ginsenoside Rb1, ginsenoside Rg1, ferulic acid, and Berberine at concentrations (in mg/mL) of 0.8045, 0.8765, 0.6932, 0.5816, 0.3993, 2.4183, and 1.0424, respectively. Recent studies have shown that emodin, one of the bioactive constituents of Radix Polygoni Multiflori, could reduce the cell death in rat cortical neurons by upregulating Bcl-2 via the estrogen receptor (ER) and phosphatidylinositol-3-kinase (PI3K) pathway [19]. Stilbene glucoside, another major extract of Radix Polygoni Multiflori, could improve the cognitive impairment by reducing oxidative stress and increasing the activity of choline acetyl transferase (ChAT). The active ingredient of Radix Ginseng Rg1 could significantly reduce phosphorylation of tau protein [20]. Li et al. established an AD model by injecting D-galactose and A*β*_1–42_ and found Rgl affects tau protein phosphorylation by inhibiting the activity of CDK5 in hippocampal neurons [21]. The active ingredient of Rhizoma Acorus could inhibit cholinesterase activity so as to delay neurodegeneration and improve cognition impairment [22]. Berberine (BR), one of the main extracts of Rhizoma Coptis, may improve the spatial memory in AD rats [23]. Durairajan found that Berberine regulates tau phosphorylation by Akt/GSK-3 and thus plays a role in protecting neurons [24]. It had been confirmed that HYD improves the ability of spatial learning and memory in various models of AD [25–27]. Although the specific mechanism of how traditional Chinese medicine affects AD model rats was not fully elucidated, our study sets the foundation for future investigation into the role of tau protein phosphorylation in the progression of AD. Collectively, the traditional Chinese medicine HYD has numerous biological effects that together may represent an important component for the development of an effective treatment regimen for patients with AD.

## Supplementary Material

Figure S1: Chemical composition analysis of HYP by HPLC. Peaks shown in numbers indicate 1, Emodin; 2, stilbene glycoside; 3, ginsenoside Re; 4, ginsenoside Rb1; 5, ginsenoside Rg1; 6, ferulic acid and 7, Berberine.
