# Supplementary figures and images for: Traditional Chinese Medicine Huannao Yicong Decoction Extract Decreases Tau Hyperphosphorylation in the Brain of Alzheimer's Disease Model Rats Induced by Aβ 1–42
Source: Evid Based Complement Alternat Med. 2016 Nov 29;2016:6840432. doi: 10.1155/2016/6840432 (PMC5153479; doi:10.1155/2016/6840432)

Relative Abundance

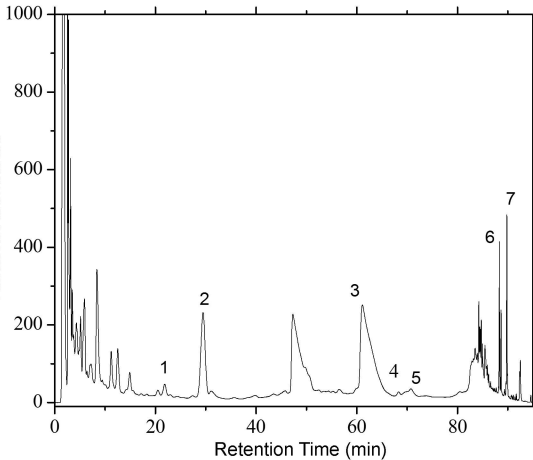

Supplement: Supplementary file 1 — Figure S1: Chemical composition analysis of HYP by HPLC. Peaks shown in numbers indicate 1, Emodin; 2, stilbene glycoside; 3, ginsenoside Re; 4, ginsenoside Rb1; 5, ginsenoside Rg1; 6, ferulic acid and 7, Berberine. [file 6840432.f1.pdf]
